# The Unfinished Reconstructed Nature of the Last Universal Common Ancestor

**DOI:** 10.1007/s00239-024-10187-8

**Published:** 2024-07-18

**Authors:** Luis Delaye

**Affiliations:** Departamento de Ingeniería Genética, Cinvestav Unidad Irapuato, Km 9.6 Libramiento Norte Carretera Irapuato-León CP. 36824, Irapuato, Gto. Mexico

**Keywords:** Early evolution of life on earth, Comparative genomics, Astrobiology

## Abstract

The ultimate consequence of Darwin’s theory of common descent implies that all life on earth descends ultimately from a common ancestor. Biochemistry and molecular biology now provide sufficient evidence of shared ancestry of all extant life forms. However, the nature of the Last Universal Common Ancestor (LUCA) has been a topic of much debate over the years. This review offers a historical perspective on different attempts to infer LUCA’s nature, exploring the debate surrounding its complexity. We further examine how different methodologies identify sets of ancient protein that exhibit only partial overlap. For example, different bioinformatic approaches have identified distinct protein subunits from the ATP synthetase identified as potentially inherited from LUCA. Additionally, we discuss how detailed molecular evolutionary analysis of reverse gyrase has modified previous inferences about an hyperthermophilic LUCA based mainly on automatic bioinformatic pipelines. We conclude by emphasizing the importance of developing a database dedicated to studying genes and proteins traceable back to LUCA and earlier stages of cellular evolution. Such a database would house the most ancient genes on earth.

## Introduction

### The Universal Ancestor: A Historical Perspective

That all life is related by common ancestry is perhaps the most simple but powerful and unifying hypothesis of all biology. Charles Darwin in his seminal work *On the Origin of Species,* foreshadowed this idea when he wrote, “probably all the organic beings which have ever lived on this earth have descended from someone primordial form into which life was first breathed” (Darwin 1859). This, rather poetical sentence, implies that life arose only once on earth. This may not had been the case since life could have originated more than once. Modern research distinguishes between the first living entities (Darwin’s primordial forms) and the last universal common ancestor of extant life (Gogarten and Olendzenski 2002). The entity proposed by Darwin is better described by the concept of FUCA standing for First Universal Common Ancestor (Prosdocimi et al. 2019).

How can we unravel the nature of the universal ancestor? Charles Darwin intuited that shared traits among living beings are the result of common ancestry: “Nevertheless all living things have much in common, in their chemical composition, their germinal vesicles, their cellular structure, and their laws of growth and reproduction” (Darwin 1859). Modern inferences on the nature of the universal ancestor are based on the same basic premise: universally conserved traits, are likely to descent from the universal ancestor. Since early 20th century biochemists suspected the universality of biochemistry [Kluyver and Donker (1926); reviewed in Singleton and Singleton (2017)] and molecular biology has confirmed Darwin’s intuition that extant life is related by common ancestry.

The universality of the genetic code stands as one of the strongest pieces of evidence for shared ancestry among all extant cells. However, as argued here, the nature of the universal ancestor remains an active area of research. The difficulty to infer the biology of the universal ancestor derives from the antiquity of its existence and the complexity of the evolutionary process connecting the universal ancestor to its descendants.

Our understanding of the last universal common ancestor is intimately linked to how we depict the phylogenetic relationships of all life on earth in the tree of life. The first image of a universal tree of life is attributed to Ernest Haeckel in his *Generelle Morphologie der Organismen* (1866). In this work, Haeckel published a plate “Monophyletischer Stammbaum der Organismen” (Monophyletic Family Tree of Organisms), were he attempted to picture the genealogical relationships between all organisms (Dayrat 2003). Notably, he created a new phylum called Protista to categorize microscopic organisms, including bacteria.

At the base of his genealogical tree, where the branches of Animalia, Plantae and Protista converged, Haeckel positioned the Monera. We envisioned them as the direct descendants of a single origin of life by spontaneous generation. Haeckel defined the first forms of Monera as *extremely simple and completely homogeneous and structureless organisms* [reviewed in Dose (1981)]. Interestingly, despite favoring the hypothesis of the monophyly of all life, Haeckel did not entirely exclude the possibility that different phyla had independent origins by spontaneous generation [reviewed in Dayrat (2003].

The turn of the 20th century saw microorganisms gaining increasing importance in our understanding of earth’s life diversity. For example, in 1938, Copeland proposed elevating Haeckel’s Monera to its own kingdom due to their lack true nuclei and their status as “the comparatively little modified descendants of whatever single form of life appeared on earth” (Copeland 1938). Following this, Stanier and van Neil (1941) further characterized the Monera kingdom, noting the absence of true nuclei, sexual reproduction and plastids.

The terms prokaryote and eukaryote were reintroduced in 1962 by Stanier and van Niel (1962) to define the concept of a bacterium. These terms were originally proposed by Chatton (1925) in an article about the evolution of primitive flagellated protozoa. However, the work *Titres et Travaux Scientifiques* (Chatton 1938) is most commonly cited as the origin of these terms. Although the prokaryote-eukaryote dichotomy was introduced by Stanier and van Neil (1962) as a way to classify cells based on its organization, it was adopted by microbiologist as a phylogenetic distinction legitimating the existence of the kingdom Monera to accommodate bacteria. This, in turn, influenced ecologist Whittaker who proposed his five-kingdoms classification scheme in 1969, encompassing Monera, Protista, Fungi, Plantae and Animalia (Whittaker 1996).

The prokaryote-eukaryote dichotomy and the five-kingdom classification scheme, while rich in biological knowledge, were not built upon a single, unifying homologous character common to all life forms from which the evolutionary relationships between the species could be inferred.

A dramatic shift occurred in 1977 when Woese and Fox compared fragments of the SSU rRNA and discovered that life has three main lines of descent (Woese and Fox 1977a). In their seminal paper, Woese and Fox argued that archaebacteria (now archaea), bacteria and the *urkaryotes* (the nucleocytoplasmic component of eukaryotes) are the main lines of descent representing “the overall phylogenetic structure of the living world”. Woese and Fox emphasized their evolutionary significance by naming these lineages as *urkingdoms*. They clearly exposed in their article that the prokaryote–eukaryote dichotomy is not a phylogenetic distinction although it is often treated as if it were. And their work showed that phylogenetic structure of the biosphere is tripartite and not bipartite as the prokaryote–eukaryote dichotomy suggested.

According to Woese and Fox (Woese and Fox 1997a, b) eukaryotes likely evolved from cells having a prokaryotic-like nature which in turn originated from even simpler entities named *progenotes*. The *progenote* was defined as a primitive entity that “had not yet completed evolving the link between genotype and phenotype” (Woese and Fox 1977b). This proposal aligns well with the RNA world hypothesis [reviewed Higgs and Lehman (2015)] which suggests that a single type of RNA molecule could have embodied phenotype and genotype. This characteristic positions the *progenote* closer to the potential origin of life (Gogarten and Olendzenski 2002).

These discoveries paved the way for Fitch and Upper (1987) proposal of the *cenancestor* defined as “the most recent ancestor common to all organisms that are alive today (cen-, from the Greek kainos, meaning recent, and koinos, meaning common)”. Lazcano et al. (1992) later argued that the *cenancestor* was likely closer in complexity to extant prokaryotes than to *progenotes*. A proposal that was based on shared traits (homologous gene sequences) between archaea, bacteria and eukarya.

The proposal that the *cenancestor* had a bacterial-like nature gained momentum by the discovery that universally conserved paralogous genes supported a bacterial root in the universal tree of life (Gogarten et al. 1989; Iwabe et al. 1989; Gribaldo and Cammarano 1998). Based on this rooting of the tree of life, Woese et al. (1990) proposed to classify all life on earth on three domains: Archaea, Bacteria and Eucarya where the latter two share a most recent common ancestor (Woese et al. 1990). With these advances, evolutionary biologist felt closer to deciphering the structure of the universal tree of life.

With the availability of the first complete cellular genome sequences, those of the parasitic bacteria *Mycoplasma genitalium* and *Haemophilus influenzae*, Mushegian and Koonin (1996) attempted to identify the minimal gene set essential for life. They reasoned that genes conserved between these two distant bacterial lineages would tend to be essential to any cell. They identified 256 conserved proteins between the two bacteria, most of which also had homologs in eukarya and archaea. However, there were important exceptions. Notably, seven key proteins involved in DNA replication, including the main replicative polymerase that lacks homology between bacteria and archaea/eukaryotes.

Based on the lack of shared homology between the DNA replicative polymerase across the tree of life, Mushegiand and Koonin (1996) speculated that the last common ancestor of the three domains (Archaea, Bacteria and Eucarya) may have possessed an RNA genome. However, Becerra et al. (1997) challenged this proposal. They presented three main arguments: (i) the level of sequence conservation found in universally conserved large proteins indicate that the genetic apparatus of the *cenancestor* already had high fidelity, an unlikely scenario for RNA-based systems; (ii) sequence and biochemical analysis of a ribonucleotide reductase from *Pyrococcus furiosus* suggests homology to their bacterial and eukaryal counterparts; and (iii) their analysis was performed before any archaeal and eukaryal genome was completely sequenced and therefore should be considered preliminary. Becerra et al. (1997) also argued that comparing the genomes of *M. genitalium* and *H. influenzae*, both parasitic bacteria, to infer the gene content of the cenancestor is skewed. These streamlined genomes, due to its parasitic lifestyle, have been particularly affected by secondary gene losses.

As more complete genomes sequences were available it was expected that the majority of the gene phylogenies supported the three domain classification of life. However, this was not the case. Many protein families exhibited phylogenies were archaea, bacteria and eukarya did not form distinct ancestral lineages (monophyletic groups).

Several non-exclusive explanations could account for these unexpected phylogenetic patterns: undetected paralogy (gene duplication followed by differential gene loss), phylogenetic artifacts like long-branch attraction or lack of clear phylogenetic signal, and/or horizontal gene transfer. To accommodate the observed phylogenetic data to evolutionary theory, Ford Doolittle suggested that in the long run the history of life is dominated by horizontal gene transfer, in particular among prokaryotes (Doolittle 1999). This implied that it would be challenging (if not impossible) to infer the gene content of the *cenancestor* simply by identifying homologous genes between the three domains of life.

Building on the notion that horizontal gene transfer was even more prevalent during the early evolution of life, Carl Woese proposed that the universal ancestor was better described by a genetic annealing model (Woese 1998). In this model, lateral gene transfer and not vertical inheritance dominated the evolutionary dynamic among *progenotes*. Woese suggested that different components of cells became refractory of lateral gene transfer (“crystallized”) at different times, with the translation apparatus being among the first. Over time, cellular lineages gradually emerged from this communal ancestor. Consequently, the universal tree of life is not organismal at is base.

According to this view, it makes no sense to infer the biological properties of the *cenancestor* because such an entity did not exist as a cell. Rater this entity existed as a “process characteristic of a particular evolutionary stage”. Building on this concept, Line (2002) proposed the term Last Common Community (LCC) to emphasize that the universal ancestor is better described by a gene-sharing community of cells.

Around this time, Patrick Forterre proposed the acronym LUCA (Last Universal Common Ancestor) as a synonym of the *cenancestor* (Forterre 1997). This name has successfully conquered the literature ever since. In his work, Forterre argued that LUCA was likely more complex than the version envisioned by Mushegian and Koonin (1996). He proposed that a combination of comparative genomics, molecular phylogeny and comparative biochemistry could be used to further define LUCA’s biological characteristics.

A few years later, Leipe et al. (1999) suggested that DNA may have evolved twice, once in the branch leading to bacteria and another time in the branch leading to archaea and eukarya. This proposal was based on a detailed analysis of the homology relationships of the different components of the replication apparatus between archaea, bacteria and eukarya. They proposed that LUCA had a mixed RNA/DNA genetic system with reverse transcriptase playing a central role (Leipe et al. 1999).

More recent research by Koonin et al. (2020) challenges the proposal of a retrovirus-like genetic system in LUCA. This is based on the fact that: (i) there are several components of the replication apparatus that are universally conserved (such as the clamp loader ATPase, the sliding clamp PCNA and the ssDNA-binding proteins); (ii) the relative complexity inferred for LUCA (already capable of ribosome mediated protein synthesis); and (iii) the discovery of the homology between the archaeal replicative DNA polymerase PolD and the transcriptase (Sauguet et al. 2019).

The homology of these two enzymes (PolD and the transcriptase) allowed Koonin et al. (2020) to propose a model for the evolution of the main replicative DNA polymerase within the context of a DNA-genome-based LUCA. Their model suggests that LUCA possessed a replicative DNA polymerase ancestral to PolD. This ancestral enzyme, in turn, likely evolved from a replicative RNA polymerase that emerged during the RNA–protein world. Koonin et al. (2020) proposed that this RNA polymerase likely originated from a homolog of the RIFT barrel found in EF-Tu like translation factors and ribosomal protein L3 (Koonin et al. 2020).

### The Inference of LUCA in the Age of Genomics

With the availability of complete archaeal genomes, Kyrpides et al. (1999) pioneered the reconstruction of the genome of LUCA. Their approach, for the first time, incorporated complete genomes from all three domains of life, but also extended beyond them. They establish a straightforward criterion for assigning a protein/function to LUCA: if a protein in the archaeal domain had homologs in either bacteria or eukaryotes, it was presumed to be present in LUCA.

Kyrpides et al. (1999) identified a total of 324 proteins in *Methanococcus jannaschii* with at least one homolog in bacteria and eukarya. These 324 proteins were classified in 246 universally distributed functions. Based on their analysis, LUCA was “possibly a complex organism a complex organism, with most “structural” components of metabolic pathways and some genetic information processing in place, but without “regulatory” elements such as replication, cell division, and intracellular regulation”. In their interpretation LUCA possessed a metabolism similar to modern cells, but with a transcription system more like that of archaea.

**Fig. 1:**
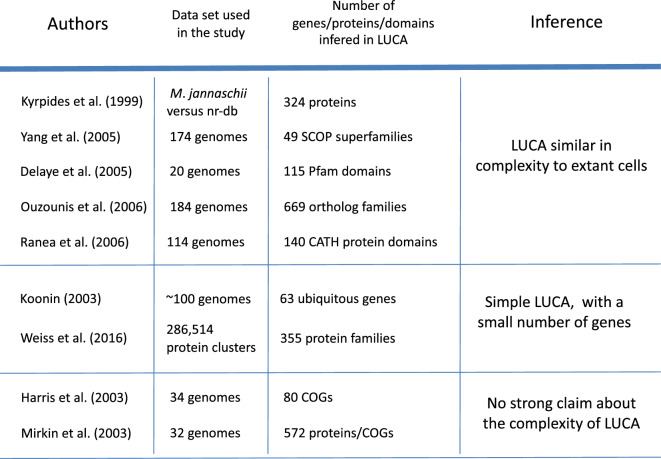
Different studies assign varying number of proteins to LUCA. This variation stems from differences in the genome sets and methodologies employed by each study. Some of these studies suggest that LUCA possessed a complexity comparable to extant prokaryotes. Conversely, others propose a simpler entity, more akin to *progenotes* and closer to the origin of life. It is important to note that the list of studies is not compressive

As more complete genome sequences became available, researchers attempted new inferences about the gene complement of LUCA (Fig. 1). However its nature remained a topic of debate (Fig. 1). Some researchers, including Kyrpides et al. (1999), Delaye et al. (2005), Yang et al. (2005), Ouzounis et al. (2006) and Ranea et al. (2006) argued for a LUCA similar in complexity to existing prokaryotes. Others, as previously mentioned, proposed a much simpler entity (Koonin 2003; Weiss et al. 2016a, b). Still others, like Harris et al. (2003) and Mirkin et al. (2003) did not make any strong claim about LUCA’s complexity. As evidence by recent studies (Weiss et al. 2016a, b; Carpitto et al. 2022) the debate surrounding LUCA’s complexity continues.

Accurately inferring the gene complement of LUCA hinges critically on two factors: identifying orthologous proteins inherited from LUCA billions of years ago, and accounting for the frequency of secondary gene losses and HGT through evolution. Different attempts to identify which genes were coded by LUCA address these challenges using diverse methodologies.

How to identify the most reliable set of genes dating back to LUCA? One of the simplest approaches involves identifying “universally” across a sample of bacterial, archaeal and eukaryaotic genomes. Koonin (2003) employed this method, comparing approximately 100 genomes and identified 63 ubiquitous proteins. The vast majority of these proteins function in the translation apparatus and only a few involved in transcription or DNA replication/repair. While it’s highly likely these proteins were inherited from LUCA, the possibility remains that some originated before LUCA and became universal through HGT.

To address the possibility that some of the proteins become universal through HGT, Harris et al. (2003) searched for “universally” conserved Cluster of Orthologous Groups of proteins (COGs) within a set of 34 genomes exhibiting three domain phylogenies. In this phylogenies archaea, bacteria and eukarya appear as monophyletic groups (meaning there is no evidence of inter-domain HGT). Using this stricter criterion, the number of COG protein families with these properties is small. Only 50 out of 80 universally conserved COGs showed three domain phylogenies. As expected, most of these COGs are associated with ribosomal functions, with a few involved in transcription, DNA replication and other processes.

The above approaches described above identify a set of genes that very inherited from LUCA. However, these genes alone are insufficient to sustain a living cell. In all likelihood, LUCA’s genome contained additional genes. One possibility is that some of the genes that were in the LUCA’s genome are not “universal” anymore (conserved across a diverse sample of genomes representing life’s biodiversity). This lack of universality could be due to secondary gene losses or non-orthologous gene displacement (Becerra et al. 1997; Koonin 2003).

To address the confounding factors of HGT and secondary gene loss, Mirkin et al. (2003) developed an algorithm to reconstruct a more complete picture of LUCA’s encoded genes. This approach involved mapping species protein distribution within COGs onto a phylogenetic tree that presumably reflects accurate organism relationships (i.e., a species tree). They employed a parsimony algorithm to reconstruct the evolutionary history of each COGs protein family along the species tree, accounting for both secondary gene losses and HGT events. Their analysis suggested that if HGT events are roughly as frequent as secondary gene losses, most of the essential biochemical pathways are reconstructed in LUCA. Using this approach, they assigned approximately 572 genes to LUCA’s genome.

In a similar approach, Ouzounis et al. (2006) employed all-against-all BLAST comparisons followed by a clustering algorithm to identify orthologous protein families and utilized a parsimony-based method to reconstruct ancestral states, considering both HGT and secondary gene losses. By this approach, Ouzounis et al. (2006) assigned 669 ortholog families to LUCA.

The accuracy of above methods hinges on several factors. First, the methods relay on the correct parametrization of gene losses and HGT events through evolutionary history. Second, the topology of the phylogenetic tree (the species tree) used to map protein family evolution is critical. Third, this approach depends on the accuracy of COGs to have correctly identified all orthologs. Finally, these analyses do not consider the individual evolutionary histories of each COG family.

Proteins often evolve through domain fusion (Orengo and Thornton 2005). These domains are often considered the building blocks and evolutionary units of proteins. To gain a domain centric view of LUCA’s proteome, Delaye et al. (2005) identified protein families shared across a set of 20 archaeal, bacterial and eukariotic genomes. They then assigned to LUCA those protein families exhibiting the same domain structure in *Escherichia coli*, *M. jannaschii* and *Sacharomyces cereviciae* as identified by the Pfam database (Mistry et al. 2021). This analysis yielded a set of 115 Pfam domains inferred to be inherited from LUCA.

Shared 3D structures among Pfam families often suggest homology, making structure-based approaches well-suited for identifying distant homologs, provided the proteins share similar enough structures to suggest common ancestry. For example, Ranea et al. (2006) employed an algorithm to compare structural similarities between protein domains and complemented this approach by profile-based methods, functional information and expert curation to determine homology. Similarly, Yang et al. (2005) utilized profile-based to investigate the phylogenetic distribution of homologous superfamilies, defined at the 3D-level in SCOP database. These studies did not assign large number of proteins (or protein domains) to LUCA. This can be partially explained by the relatively small number of proteins with known 3D structure at the time. As Yang et al. (2005) noted, only around 60% of proteins per genome had assigned domain superfamilies.

These different approaches offer distinct strengths and weaknesses. While the tertiary structure of proteins tends to be more conserved than their primary structure, making 3D-level searches more sensitive to detect distant homologs (Brenner et al. 1998; Yang et al. 2005; Ranea et al. 2006), analyses that consider protein phylogenies (derived from primary structure information) can distinguish orthologs from paralogs or even xenologs. This distinction allows for more precise inferences about LUCA’s gene complement (Harris et al. 2003; Mirkin et al. 2003; Ouzounis et al. 2006). Consequently, the sets of proteins inferred to be by LUCA in different studies show incomplete overlap.

**Fig. 2:**
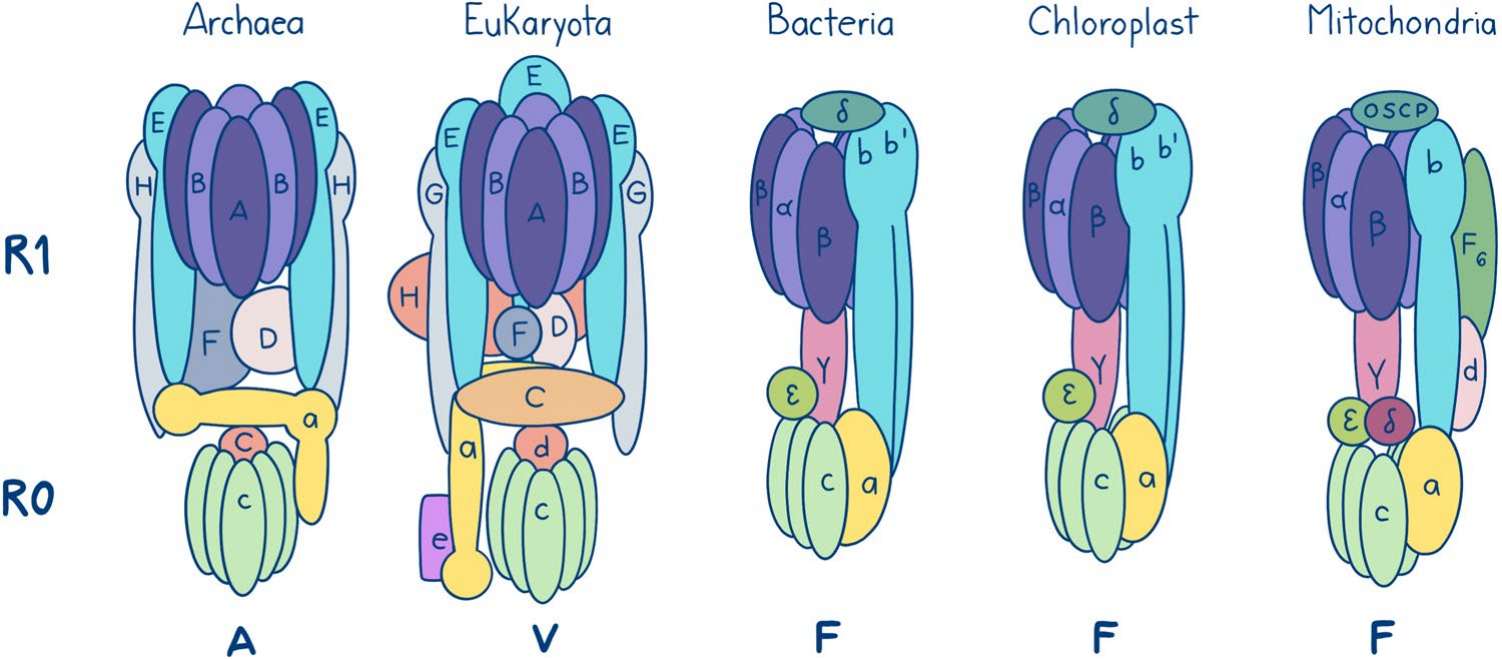
Structure and protein subunits of ATP synthase from F- and A/V-types. Figure based on Mahendrarajah et al. (2023)

A case in point is the contrasting predictions regarding the identification of ATP synthase protein subunits present in LUCA. ATP synthase, a multiprotein complex crucial for energy metabolism, comprises two subunits: Ro and R1. Each subunit is further composed of several distinct proteins (Fig. 2). Ro is the hydrophobic segment embedded in the cell membrane, while R1 is the hydrophilic, peripheral component. ATP synthases are categorized into F-, A-, and V-types. A-type and V-type enzymes share a closer evolutionary relationship than either does with F-type ATPases. While exceptions exist, bacteria predominantly possess F-type ATPases, while archaea typically have A-type. Eukaryotes, due to their origins through symbiogenesis, contain a combination of F- and A/V-type ATP synthases.

This enzyme has a complex evolutionary history that predates LUCA (Mahendrarajah et al. 2023). Notably, the R1 subunit possesses a structure that includes three catalytic and non-catalytic protein subunits forming a hexamer. These subunits have distinct designations in F-type (F1-alpha, non-catalytic; F1-beta, catalytic) and A/V-type (A, catalytic; B, non-catalytic) ATPases. As mentioned previously, the catalytic and non-catalytic subunits arose from a gene duplication event before LUCA and have even been used to root the tree of life (Gogarten et al. 1989). Recent analyses suggest that the divergence between F- and A/V-type ATPases occurred very early in life's history, possibly even coinciding with the existence of LUCA (Mahendrarajah et al. 2023). This implies that LUCA might have encoded for both types of ATP synthases.

How effective are the methods described above in identifying the protein components of ATP synthase in LUCA? As Table 1 shows, different methods identified varying sets of ATP synthase protein subunits as potentially present in LUCA. As expected, methods that accounted for horizontal gene transfer (HGT) and/or secondary gene losses, like Mirkin et al. (2003), assigned the largest number of protein subunits to LUCA. Conversely, highly stringent methods requiring protein families to show no evidence of HGT assigned the fewest subunits (Harris et al. 2003; Weiss et al. 2016a, b). Similarly, approaches reliant on pre-determined 3D structures also yielded a low number of inferred subunits (Ranea et al. 2006). Clearly, no single method successfully identified all protein subunits as part of LUCA's ancestral complement. By combining the results from different methods, we may provide a more complete picture of the ATP synthase coded by LUCA.

**Table 1:** Different methods identify diverse sets of ATP synthase protein subunits as potentially inherited from LUCA

Authors	F1-beta, A1/V1-A (catalytic)	F1-alpha, A1/V1-B (non-catalytic)	A1/V1-D	Fo-c, A1/V1-c	Fo-a, A1/V1-a/I	Fo-delta	Fo-epsilon	Fo-gamma
Kyrpides et al. (1999)	yes	yes	yes		yes			
Harris et al. (2003)				yes				
Mirkin et al. (2003)	yes	yes		yes	yes	yes	yes	yes
Delaye et al. (2005)	yes	yes						
Ouzounis et al. (2006)	yes	yes			yes			
Ranea et al. (2006)	yes							
Weiss et al. (2016)					yes			

Crapitto et al. (2022) reached a similar conclusion through a more compressive analysis. They compared protein predicted to be coded by LUCA from eight independent studies. Remarkably, none of the eight sets exhibited substantial overlap. At best, two studies showed a Jaccard’s similarity score of only 0.27 (where 0 represents no similarity and 1 indicates complete overlap). However, several studies performed moderately to well in a test designated to assess the level of agreement between individual predictions and the consensus of the remaining seven studies (inter-rater tests). Based on these findings, the authors propose that the combined sets of proteins from all eight studies, rather than any single set alone, provides a more accurate representation of LUCA’s proteome (Crapitto et al. 2022).

### Phylogenetic Approach to LUCA’s Gene Complement: Mitigating Biases

One of the most recent and influential attempts at reconstructing LUCA’s gene complement was by (Weiss et al. 2016a, 2016b). As with previous efforts, new knowledge about the tree of life fueled a novel approach to inferring LUCA’s nature. Recent phylogenetic analyses suggested that eukaryotes emerged within the archaea (Williams et al. 2013; Raymann et al. 2015; Eme et al. 2023). This positioned LUCA as the direct ancestor of archaea and bacteria. Based on this revised phylogenetic scheme, Weiss et al. (2016a, b) searched for protein families whose phylogenetic trees displayed bacteria and archaea as monophyletic groups (with eukaryotes excluded from the analysis). To minimize the possibility that the presence of these protein families in bacteria or archaea were the result of inter-domain HGT, they imposed the criteria that both bacteria and archaea needed to be represented by at least two higher taxa, each containing at least two species (some exceptions were made for archaeal groups represented by a single high taxa).

Analyzing 286,514 protein families across 1,981 prokaryotic genomes (encompassing 13 archaeal and 23 bacterial groups), they identified 355 protein families that meth their criteria. This reconstruction revealed a proteome rich in metabolic enzymes, providing insights into the environment inhabited by LUCA. Their analysis suggests LUCA was an anaerobic organism capable of CO_2_ and N_2_ fixation, utilized the Wood-Ljungdahl pathway, relied on H_2_ for energy and likely thrived in a hydrothermal setting.

The proposal by Weiss et al. (2016a, b) depicts a universal ancestor that is clearly more complex than a *progenote* because it already coded for proteins and had a relatively complex metabolism. These authors suggested that LUCA was “half-alive” because his metabolism was dependent on geochemistry. While the concept of “half-alive” is not defined, from the reading of the article it is possible to interpret that what the authors meant is that the metabolism of LUCA was primitive and closer to an autotrophic origin of life. Which can be interpreted that LUCA was simpler in complexity to extant prokaryotes.

The conclusions reported by Weiss et al. (2016a, b) were rapidly challenged by Gogarten and Deamer (2016). Perhaps most importantly, these authors indicated that Weiss et al. (2016a, b) used a methodology that favors the inclusion of genes that “have a limited distribution and utility in today’s organisms”. The argument goes as follows: on the first place, false positives can be assigned to LUCA if a gene is horizontally transferred between archaea and bacteria before the split of the two taxonomic groups in the receiving cellular domain or if the gene was transferred to one such group and then horizontally transferred to the other group; and on the second place there will be many false negatives because of the frequency of HGT along evolution (many genes present in LUCA will be filtered-out by their methodology). As a result, the list of genes assigned to LUCA will be biased towards enzymes shared by HGT between prokaryotes adapted to the same environment. Thermophilic environment is one such case.

The enzyme reverse gyrase can illustrate the case for a false positive. Reverse gyrase is the only enzyme found ubiquitously in hyperthermophilic organisms and absent in mesophiles (Forterre 2002). Weiss et al. (2016a, b) predicted that LUCA encoded reverse gyrase, suggesting a hyperthermophilic nature for the last universal common ancestor. However, recent phylogenetic analyses of reverse gyrase by Catchpole and Forterre (2019) did not recover the monophyly of bacteria and archaea. Instead, the phylogenetic analysis showed that reverse gyrase has undergone several interdomain horizontal transfer events.

Why the phylogenetic analysis of Weiss et al. (2016a, b) differs from that of (Catchpole and Forterre 2019)? Two key factors contribute to the discrepancies. On the first place, Catchpole and Forterre (2019) used a significatively larger dataset of enzymes (97 versus 376); on the second place, the internal branch separating bacteria from archaea in the tree inferred by Weiss et al. (2016a, b) is particularly small. This contrast with typical trees inferred from other molecular markers such as 16S rRNA, *rpoB* (encoding the RNA polymerase *β*-subunit), and translation elongation factor G, where bacteria and archaea are separated by a longer and well-supported internal branch. As Catchpole and Forterre (2019) suggest, requiring genes to exhibit phylogenies with longer and well-supported branch separating bacteria and archaea could have minimized false positives in the analysis by Weiss et al. (2016a, b).

An additional approach to reduce false positives would be to increase the number of required higher taxa within archaea and bacteria for assigning a gene to LUCA. While the phylogenetic approach followed by Weiss et al. (2016a, b) to infer LUCA’s nature is promising, more meticulous efforts are needed.

### Mapping LUCA’s legacy: Building a Database of Earth’s Most Ancient Genes

Despite the abundance of online databases for storing, classifying and describing life’s molecules, a dedicated resource specifically for universally conserved genes potentially dating back to LUCA and earlier stages of cellular evolution remains scare. LUCApedia stands as the only exception (Goldman et al. 2013). This database integrates the results from six different studies on LUCA’s gene complement into a MySQL database (Harris et al. 2003; Mirkin et al. 2003; Delaye et al. 2005; Yang et al. 2005; Wang et al. 2007; Srinvasan and Morowitz 2009). As indicated by Carpitto et al. (2022), a new version of LUCApedia is expected to be available soon.

Ideally, a database of genes likely descended from LUCA should be built through a combination of bioinformatic pipelines and manual curation (Crapitto et al. 2022). As exemplified here, no single bioinformatic pipeline is perfect: some might miss genes from LUCA while others might include false positives. Therefore, automatic efforts should be complemented by careful hands-on molecular analyses.

The first step would involve a meticulous selection of archaeal and bacterial genomes. This selection should represent the major branches of life while excluding highly reduced genomes. Bioinformatic pipelines could then be used to identify an initial set of protein families potentially inherited from the last universal common ancestor. These families could then be manually analyzed to reconstruct their evolutionary history and assess their candidacy for inclusion in LUCA’s gene repertoire. The resulting set of genes/proteins assigned to LUCA, along with their phylogenies and evolutionary histories, could be used to evaluate the likelihood of hypothesis to about the nature of our universal ancestor. Such a database would house the most ancient genes on earth, supporting the hypothesis of universal common ancestry first proposed by Charles Darwin.
